# Rapid tannin profiling of tree fodders using untargeted mid-infrared spectroscopy and partial least squares regression

**DOI:** 10.1186/s13007-021-00715-8

**Published:** 2021-02-06

**Authors:** Jordi Ortuño, Sokratis Stergiadis, Anastasios Koidis, Jo Smith, Chris Humphrey, Lindsay Whistance, Katerina Theodoridou

**Affiliations:** 1 Institute for Global Food Security, Queen’s University Belfast, Belfast, BT9 5DL Northern Ireland, UK; 2 Department of Animal Sciences, School of Agriculture, Policy and Development, University of Reading, New Agriculture Building, Earley Gate, P.O. Box 237, Reading, RG6 6EU UK; 3 Organic Research Centre, Trent Lodge, Stroud Road, Cirencester, Gloucestershire, GL7 6JN UK; 4 Present Address: MV Agroecological Research Centre, Moinhos de Vento, 7750-217 Espirito Santo, Portugal

**Keywords:** Condensed tannins, Chemometrics, Tree fodders, Ruminant nutrition, Silvopastoralism, Willow, Oak, Maple

## Abstract

**Background:**

The presence of condensed tannins (CT) in tree fodders entails a series of productive, health and ecological benefits for ruminant nutrition. Current wet analytical methods employed for full CT characterisation are time and resource-consuming, thus limiting its applicability for silvopastoral systems. The development of quick, safe and robust analytical techniques to monitor CT’s full profile is crucial to suitably understand CT variability and biological activity, which would help to develop efficient evidence-based decision-making to maximise CT-derived benefits. The present study investigates the suitability of Fourier-transformed mid-infrared spectroscopy (MIR: 4000–550 cm^−1^) combined with multivariate analysis to determine CT concentration and structure (mean degree of polymerization—mDP, procyanidins:prodelphidins ratio—PC:PD and cis:trans ratio) in oak, field maple and goat willow foliage, using HCl:Butanol:Acetone:Iron (HBAI) and thiolysis-HPLC as reference methods.

**Results:**

The MIR spectra obtained were explored firstly using Principal Component Analysis, whereas multivariate calibration models were developed based on partial least-squares regression. MIR showed an excellent prediction capacity for the determination of PC:PD [coefficient of determination for prediction (R^2^P) = 0.96; ratio of prediction to deviation (RPD) = 5.26, range error ratio (RER) = 14.1] and cis:trans ratio (R^2^P = 0.95; RPD = 4.24; RER = 13.3); modest for CT quantification (HBAI: R^2^P = 0.92; RPD = 3.71; RER = 13.1; Thiolysis: R^2^P = 0.88; RPD = 2.80; RER = 11.5); and weak for mDP (R^2^P = 0.66; RPD = 1.86; RER = 7.16).

**Conclusions:**

MIR combined with chemometrics allowed to characterize the full CT profile of tree foliage rapidly, which would help to assess better plant ecology variability and to improve the nutritional management of ruminant livestock.

## Background

Silvopastoral systems involve the development of agronomical multi-models in which trees and livestock share the same land. In the British Isles, silvopastoral systems generally consist of grazed pastures enriched with trees at wide spaces, which implies potential benefits both for the environment and for the animals [1]. The tree foliage, consisting of leaves and twigs, can be offered to the ruminants either by direct browsing, chopping fresh or after preservation by drying or ensiling, thus representing a nutritional supplement during certain seasons. Oak (*Quercus* spp.), field maple (*Acer camprestre*) and willow (*Salix* spp.) are local tree species in Britain and Ireland with great potential to include in silvopastoral systems, among other benefits, due to their content in condensed tannins (CTs). Also known as proanthocyanidins, CTs are the most abundant secondary metabolite of woody plants, in which can exceed 10% in foliage dry matter (DM) [2]. The reduction of bloat incidence, antiparasitic properties, improvement of nitrogen utilization and reduction of greenhouse gas emissions are the main properties that highlight the interest of livestock producers in these bioactive compounds; however, CTs may also be considered as antinutritional factors, especially when high concentrations are present in the plant material [3].

Most of the benefits attributed to the consumption of CTs by ruminants are related to their protein binding ability [4]. Nevertheless, it is challenging to establish a relationship between CTs and their biological activities due to their chemical diversity and the lack of full understanding of the mechanisms involved [5]. Chemically, CTs are high molecular weight flavonoids consisting of flavan-3-ol units. These units can be linked to form relatively small oligomeric to long polymeric chains, defined by the mean degree of polymerization (mDP). Depending on the presence (OH) or absence (H) of a hydroxyl group at C5 of the B-ring, and the spatial orientation (cis/trans) of the OH group at C3 carbon, flavan-3-ol units can be classified in 4 different types: catechin (H, trans), epicatechin (H, cis), gallocatechin (OH, trans) and epigallocatechin (OH, cis) [6]. Catechin and epicatechin yield procyanidin (PC) tannins, whereas prodelphinidins (PD) are composed of gallo- and epigallocatechin units. The concentration of tannins, mDP, and both ratio of cis:trans and PC:PD flavan-3-ol units are decisive factors in the biological activity of CTs in animals. Therefore, a comprehensive tannin profiling is necessary to understand the mechanisms of actions of these compounds and support proper management of tree foliage for animal nutrition.

One of the most popular methods for the quantification of CT in plant materials is the Hydrochloric Acid–Butanol–Acetone–Iron (HBAI) assay [2, 7]. Although it is an excellent quantitative method, it is non-specific and does not provide information regarding CT length, structure and stereochemistry. In order to obtain detailed knowledge on CT structure (including mDP, cis:trans and PC:PC ratio), thiolytic degradation followed by HPLC or LC/MS constitutes an essential method, if successfully applied both to purified extracts or dried material. These digestion and separation methods, however, are challenging to set up and time- and resource-consuming [6]. Moreover, the concentration and chemical characteristics of CTs are dependent not only on the species but also on the climate, soil characteristics, cultivars and season, which might require regular analyses to determine the foliage CT profile effectively. All these factors make infeasible the implementation of traditional wet CT analyses for silvopastoral systems, which are often managed based on traditional knowledge rather than in empirical evaluation. To overcome these problems and establish efficient evidence-based management of silvopastoral systems, novel screening methods should be developed to characterize the tannin profile in the tree species included within grasslands.

Spectroscopic techniques combined with chemometric modelling can decrease dependency on classical CT analytical methods for quantitative analyses of large numbers of samples. Indeed, multivariate calibrations successfully predicted CT concentrations by correlating near-infrared spectroscopy (NIR) with classical analytical methods (UV-spectroscopy, chromatography) in the foliage of different tree species, such as willow [8], poplars [9], aspen and birch [10]. Apart from tannin concentration, some attempts are also reported to assess CT profile (mDP, PC:PD and cis:trans ratio) by NIR in sainfoin, although with modest results (R^2^P = 0.49–0.84) [5]. Attenuated total reflection (ATR) Fourier transform mid-infrared spectroscopy (MIR) is currently considered to be one of the most promising tools to adequately characterize CTs from different botanical sources [11]. ATR-MIR is based on the absorption of an IR wave travelling through a high refractive index prismatic crystal in close contact with a sample. The absorption data is closely correlated to the vibrational intensities of the molecular bonds of chemical functional groups of samples [12]. Therefore, the MIR-infrared region (4000–400 cm^−1^) has the potential to aid with the characterization of tannins as it provides information on fundamental molecular vibrations, which are, by definition, more specific compared to the harmonic vibrations and overtone absorptions observed by NIR. Previous research on the application of MIR to both synthetic and natural (wine) tannin sources suggested specific wavelength bands related with the presence of PC and PD, as well as other bands useful to demonstrate differences between diastereoisomers (cis:trans) [11]. MIR has also been widely employed for the quantification and characterization of tannins in grapes and wine samples. Studies in the literature claimed excellent predictions for the quantification of tannins (R^2^P > 0.95) using different methods (protein precipitation, phloroglucinolysis, UV–vis) [13–15]; as well as for the estimation of mDP (R^2^C > 0.95) [13] and the percentage of galloyllation (R^2^C > 0.98) [16].

The development of portable NIR instruments for on-site data collection and analysis is far more advanced and affordable than MIR [17]. Despite improvements done during the last decade, there are technical issues that limit the applicability of on-site MIR spectroscopy in comparison with bench-top equipment. The instrument’s energy source and detector along with the lack of preprocessing of in-field data collection may affect the quality of the MIR spectra and in turn, the prediction results [18]. The identification of MIR-exclusive benefits would imply an added-value to promote the development of portable equipment to bring its advantages over NIR from the lab to the field. In this context, there is no study assessing the potential of MIR to comprehensively profile tree foliage CT. Therefore, the present study aims to investigate the suitability of MIR combined with chemometrics analysis to rapidly determine the condensed tannin concentration, mDP, PC:PD ratio and cis:trans ratio in leaves and twigs of three tanniniferous species (oak, field maple and goat willow) commonly found in silvopastoral systems.

## Results and discussion

### MIR spectra features

**Fig. 1:**
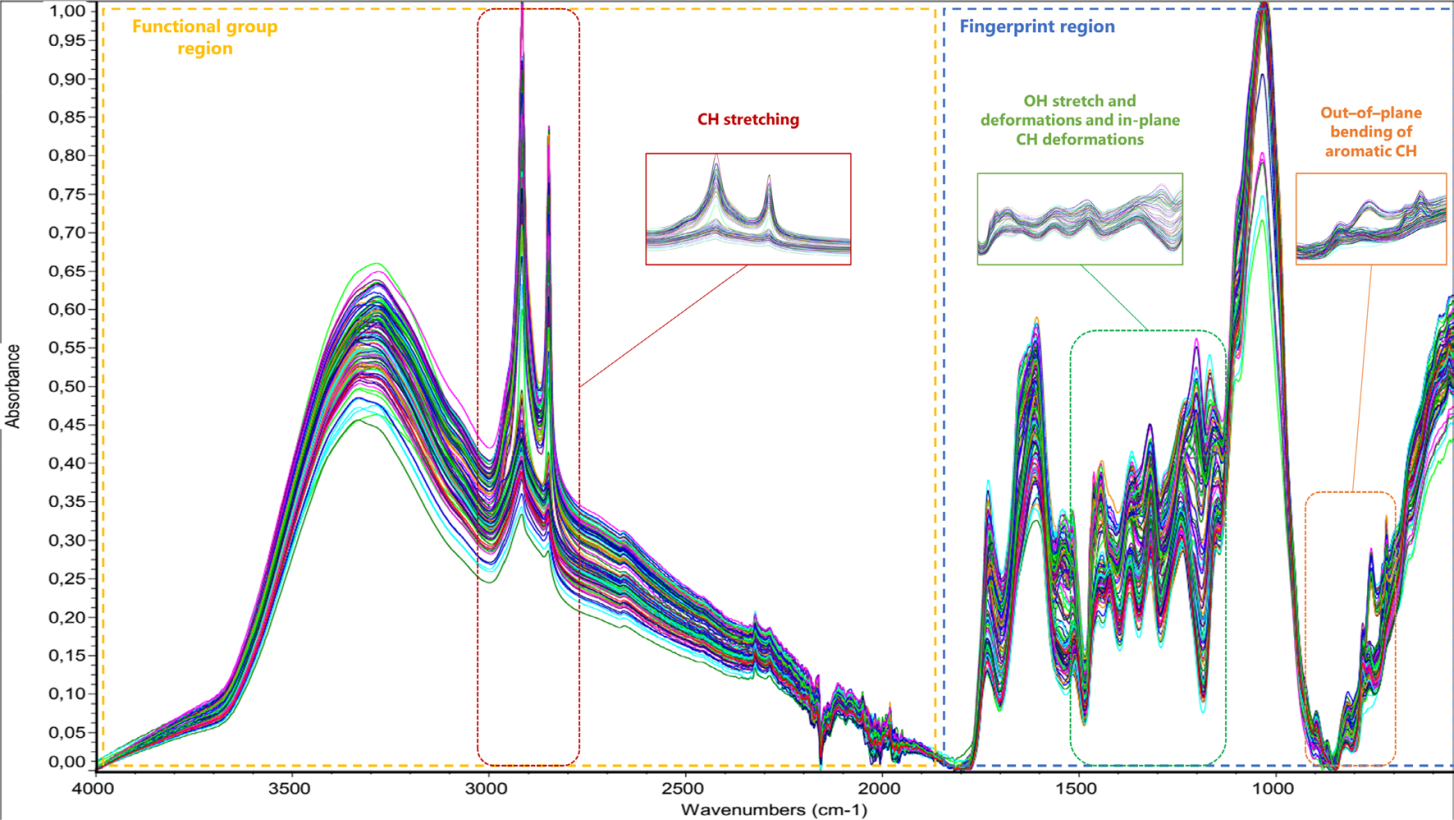
MIR spectra (4000–550 cm^−1^) of field maple, oak, and willow leaves and twigs after baseline correction and normalization scale. Principal and relevant absorption bands are highlighted and/or magnified

Figure 1 shows the foliage (leaves and twigs) spectra of the oak, maple and willow samples after the baseline correction and scale normalization. The principal absorption bands lay between 550–1800 (fingerprint region) and 2700–3000 cm^−1^ (within functional group region that generally includes from 4000 to 1800 cm^−1^). The fingerprint region is linked to bending vibrations within the molecules, representing a heterogeneous group of molecular characteristics [19]. Within this region, relevant wavelengths described previously in relation with vegetal tannins are located between 1577–1060 cm^−1^ (overlapping of OH and CH deformations) and 900–640 cm^−1^ (out-of-plane bending of aromatic -CH). The functional group region is linked to hydroxyl and alkyl stretching vibrations. Specifically, the region comprised between 2700 and 3000 cm^−1^ is related to CH_2_ and CH_3_ symmetric and asymmetric stretching vibrations. These wavelengths have been previously related to tannins [20] and to the lipid CH stretching regions in plants [12]. The division of spectra in two major groups observed in the enlarged sections observed in Fig. 1 seemed to be related to differences between leaves and twigs more than between species.

Principal Component Analysis (PCA) performed to examine the potential clustering of samples (Additional file 1: Fig. S1) showed a clear separation between leaves and twigs. However, twig samples from the three species were clustered together, whereas willow, oak and field maple leaves were kept apart from each other. Among them, willow leave samples showed higher scattering, suggesting the potential presence of outliers. Twigs from most forages are generally richer in structural carbohydrates than leaves, with lower content of lipids, proteins and tannins [21–23]. Therefore, spectra differences related to differences in major fodder constituents (including tannins, which may represent more than 10% of leaves DM) are mainly expected between leaf samples than twigs.

### Reference methods analysis

Table 1 shows a descriptive summary of the average reference method descriptive statistics of the three randomized splits of the data set, including the number of samples, mean, standard deviation, median and range. Despite the inclusion of samples from different species, plant parts (twigs and leaves) and months, the content of CT (HBAI/thiolysis) and mDP showed narrow ranges, with minimum–maximum values of 1.51/0.43–10.8/11.7 and 1.99–8.23, respectively, demonstrating similar values between species. In contrast, PC:PD and cis:trans ratio showed considerable variability, covering virtually 0 to 100% range. The random subdivision of the data set in calibration (75%) and validation (25%) samples showed comparable descriptive statistics for all parameters, suggesting an appropriate level of variation representative of the whole dataset.

**Table 1:** Descriptive statistics of calibration and validation sets

	N	Calibration set				N	Validation set			
		Mean	SD	Median	Range		Mean	SD	Median	Range
%CT (HBAI)	90	4.22	2.25	3.50	9.19	30	4.49	2.39	3.64	8.42
%CT (Thiolysis)	89	4.34	2.47	3.85	11.3	30	4.56	2.58	3.93	10.9
mDP	88	4.45	1.30	4.23	6.24	30	4.54	1.31	4.34	5.61
PC	90	66.7	28.7	63.0	82.7	30	64.6	28.7	55.9	77.4
cis	90	52.7	32.6	45.3	94.6	30	54.4	31.1	47.8	92.9

*N* number of samples, *SD* standard deviation, *%CT* content of condensed tannins (%Dry Matter) measured by HCl-butanol-acetone-iron assay (HBAI) or thiolysis-HPLC (Thiolysis), *mDP* mean degree of polymerisation, *PC* % procyanidin units (% condensed tannins), *cis* % cis units (% condensed tannins)

### PLS-R models development

Mean centering was applied as a standard procedure in spectroscopic analysis to assess the variability of the data around the mean. In our models, standard normal variate (SNV) was applied to reduce variability of spectra due to scatter, whereas a weighed Savitzky–Golay filter (polynomial degree = 2; window size = 11 points) was employed to obtain smoothed second derivative spectra, allowing both high frequency (smoothing) and low frequency signal (differentiation) noise reduction [24]. These wavelength regions were used as an initial reference for the development of the models, and further interval variable selection procedures were explored (VIP, sRatio, iPLS) to build models on fewer variables (wavelengths), which contribute to the reduction of co-linearity and redundancy.

Calibration models were developed by using PLS-R for the determination of tannin concentration (evaluated by two different methods, HBAI and thyolysis), degree of polymerization (mDP), ratio of procyantonidins:prodelphidins (expressed as the %PC of total CT content) and ratio cis:trans of diastereoisomers (expressed as %cis of total CT content). PLS-R has been successfully applied for the multivariate calibration of IR spectral data for tannin quantification and characterization from different agricultural sources [9, 10, 13]. In this study, after model optimization, the performance was compared between PLS-R and Principal Component Regression and Multivariate Linear Regression, showing PLS-R gave the best results for all parameters considered in the study (data not shown). Parameters of the PLS-R models are shown in Table 2. All models developed by PLS captured > 95% of the variance with less than 10 LVs, below the recommended limits established to avoid overfitting [(N calibration samples)/10 + 2; in our case, 11 LVs] [25]. The RMSEP was higher than the calibration bias for every model, suggesting that it was not necessary to weigh calibration as the models would not systematically over- or under-predict analyte concentrations [26]. However, all models showed fair RMSECV/RMSEC ratios (≥ 1.20), which suggests that an increase in the number of calibration samples would be recommended to improve the prediction performance [9].

**Table 2:** Summary of calibration and prediction PLS-R parameters for the tannin profile

	LV	Regions (cm^−1^)	R^2^C	RMSEC	RMSECV	R^2^P	RMSEP	RPD	RER	Cal. Bias
%CT (HBAI)	7	iPLS (1186–1454)	0.93	0.59	0.76	0.92	0.70	3.71	13.1	− 0.019
%CT (Thiolysis)	6	Main frequencies (551–1810/2700–3000)	0.87	0.89	1.08	0.88	0.93	2.80	11.5	− 0.215
mDP	6	Full range (550–4000)	0.75	0.64	0.84	0.66	0.77	1.86	7.16	− 0.015
PC	8	Fingerprint (700–1700)	0.97	4.96	6.82	0.96	5.60	5.26	14.1	0.208
cis	6	iPLS (9 regions^a^)	0.96	6.74	8.39	0.95	7.07	4.24	13.3	− 0.157

*LV* latent variables, *RMSEC/CV/P* root-mean-square error or calibration/cross-validation/prediction, *RPD* ratio of prediction to deviation, *RER* range error ratio, *%CT* content of condensed tannins (%DM) measured by HCl-butanol-acetone-iron assay (HBAI) or thiolysis-HPLC (Thiolysis), *mDP* mean degree of polymerisation, *PC* % procyanidin units (% condensed tannins), *cis* % cis units (% condensed tannins)

^a^646–673/790–846/877–904/1108–1135/1253–1280/1340–1367/1398–1425/1658–1685/1716–1743/1774–1810 cm^−1^

Overall, PLS-R models for the analysis of tannin profile parameters in the three tree species and foliage parts (leaves and twigs) considered in the study resulted in excellent prediction of PC:PD (R^2^P = 0.96; RPD = 5.26; RER = 14.1) and cis:trans (R^2^P = 0.95; RPD = 4.24; RER = 13.3) ratios, modest for both tannin quantification methods (HBAI: R^2^P = 0.92; RPD = 3.71; RER = 13.1; Thiolysis: R^2^P = 0.88; RPD = 2.80; RER = 11.5) and weak for mDP (R^2^P = 0.66; RPD = 1.86; RER = 7.16). Couture et al. [10] already demonstrated the ability of spectroscopy to develop robust multispecies models for the quantification of tannins in tree foliage, similar to those obtained for individual species. However, this is the first study determining the full tannin profile by spectroscopic techniques in different tree species. As shown, the development of multispecies calibration for the prediction of tannin characteristics reduces the influence of covariables and improves the usefulness of these techniques for on-site applications.

### Condensed tannins quantification

**Fig. 2:**
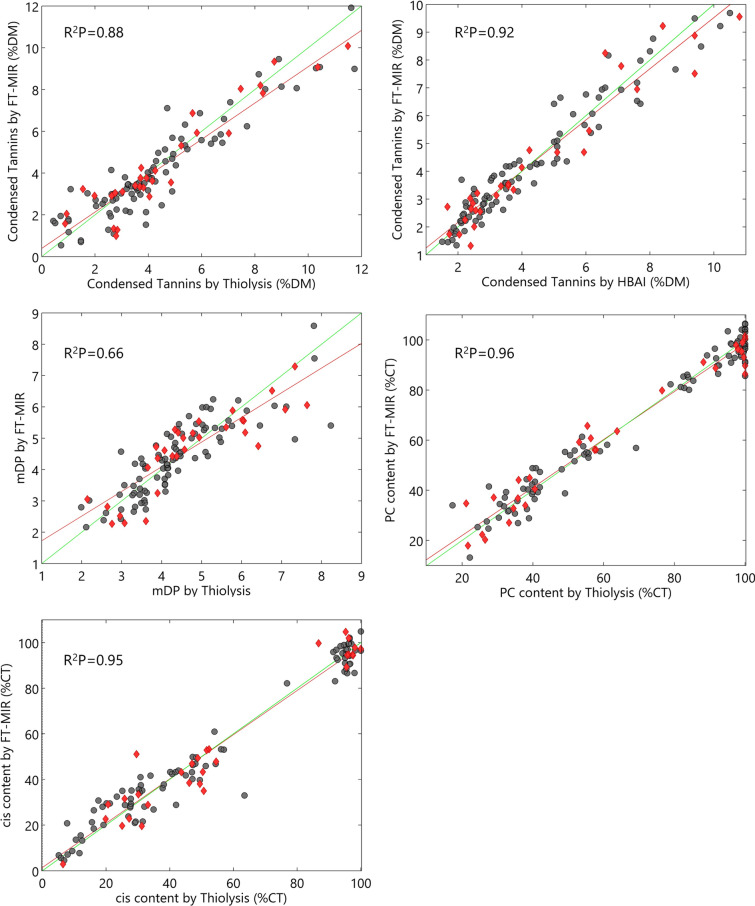
Actual vs. predicted values of the tannin profile parameters. Grey spots: calibration samples; red diamond: validation samples; green line: ideal prediction fit; red line: predicted adjusted equation; R^2^P: coefficient of prediction

The actual and the predicted values of the tannin profile parameters are presented in Fig. 2. HBAI showed higher performance than thiolysis, considering calibration (R^2^C) and prediction (R^2^P) performance (HBAI > 0.90 > Thiolysis) and RPD (HBAI > 3 > Thiolysis), even though both techniques showed RER > 10. Our results are in line with the previous quantification of tannins in dry tree foliage of aspen and paper birch (R^2^P = 0.81–0.92; %RMSEP < 10) using the HBAI method for calibration [10]. The wavelength selection showed differences in the regression models developed, which might be related to the different methods of analysis. The best performance for the calibration of MIR based on HBAI was obtained using iPLS algorithm, which suggested the region 1486–1184 cm^−1^. As commented above, wavelengths within the 1577–1060 cm^−1^ region have been suggested as relevant for tannin quantification [15], a region in which overlap OH stretch and deformations of phenols and in-plane CH deformations of aromatic compounds [27]. The most relevant wavelengths (with VIP values > 2) for HBAI were around 1450–1430 cm^−1^ (see Additional file 2: Fig. S2), a region already correlated with the presence of tree tannins foliage [15], linked explicitly to aromatic ring stretching vibrations [28]. In contrast, thiolysis-HPLC showed the best calibration performance when both main frequencies regions (1800–550 cm^−1^ and 3000–2700 cm^−1^) were employed. Beyond the fingerprint region, the 3000–2700 cm^−1^ region has also been linked to the CH stretching of tannin aromatic compounds [20], and used for their quantification [13, 29]. Indeed, VIP values > 3.5 for the thiolysis model fell within this region. Taking into account the better prediction performance, similar LVs, and the reduced number of variables employed, our results suggest a more robust and accurate tannin prediction by MIR calibrated using HBAI. Thiolysis quantification is sometimes problematic due to oxidative processes affecting reaction yields and degradation products, and higher CT yields are reported in some samples with the HBAI assay, which is generally considered the reference method for this purpose [5]. It is generally accepted a dietary CT content lower than 5 g/100 g as beneficial for animal nutrition, although this threshold value may vary depending on the tannin source and structure, diet composition and animal species [3]. Therefore, periodical screening of CT concentration, while evaluating animal performance and physiological status, can help to improve understanding on the effect of CT concentration in willow, oak and maple foliage in relation to the animal species and diet composition.

### Mean degree of polymerization

The estimation of mDP showed the weakest accuracy among CT profile parameters and was deemed not suitable for quantitative purposes (RPD < 2). The prediction coefficient obtained (R^2^P = 0.66) improved the values obtained by NIRS in sainfoin samples (R^2^P = 0.49) [5] even though it was much lower than the ones obtained in wine extracts by MIR (R^2^ = 0.96) [13]. It is interesting to highlight, along with the results of Fernández and Agosín [13], the regression models did not improve when selecting significant variables intervals, obtaining the best results when full spectra range was used. Under similar conditions, CT size (mDP) proved to affect the protein precipitation capacity [30]. However, foliage samples of oak, field maple and willow showed a small CT size within a relatively narrow mDP range (4.23–6.24). Therefore, in this case, the PC:PD and cis:trans ratio is expected to be more relevant in determining the biological activity of CTs [5].

### Tannin structure and stereochemistry

The estimation of PC:PD and cis:trans ratio obtained in tree foliage of oak, maple and willow by MIR showed higher predictive accuracy than those obtained previously in sainfoin samples by NIRS both for PC:PD (R^2^P = 0.77) and cis:trans (R^2^P = 0.84) [5]. In our PC model, the most pronounced VIP values (> 2.5) fell within 725–715, 760–750 and 1165–1155 cm^−1^ wavelengths (Additional file 2: Fig. S2). Previous studies highlighted the regions 900–640 cm^−1^ (related to both OH oscillations of aromatic alcohols and out–of–plane bending of aromatic CH) and 1225–950 cm^−1^ (related to –CH aromatic in-plane bending) for CT discrimination purposes [11]. Indeed, the hydroxylation pattern of the CT’s aromatic rings can be detected by MIR at regions between 730–780, 1000–1300 and 1520–1530 cm^−1^ [23], whereas gallocatechins (trans-OH) in comparison with catechins (trans-H) show an additional peak near 740 cm^−1^, attributable to the –CH out of plane bending of the B ring. Regarding diastereoisomers, bands in the region 800–700 cm^−1^ are attributed to a higher or lower presence of cis-isomers in flavan monomers, which include some of the regions selected by the iPLS algorithm. The dominant VIP values (> 2.5) found in the cis model (650–640 cm^−1^) fell in the out-of-plane C–OH bending of phenolic rings in a connected state. The presence of specific features for the estimation of PC:PD and cis:trans ratio within the aromatic out-of-plane bending region (900–650 cm^−1^), not overlapping with the central regions associated with carbohydrates (1480–950 cm^−1^) and proteins (1700–1485 cm^−1^) in plant foliage [12], might help to explain the higher accuracy of the MIR model in comparison with previous NIR ones, which tend to overlap [5]. A higher proportion of PC over PD results in a lower number of hydroxyl groups in the CT structure, which implies a reduced potential for hydrogen bond interactions, and thus lower binding protein capacity, inhibition of trypsin and astringency (protein precipitation) [24, 31, 32]. Similarly, the cis:trans stereochemistry of the monomeric subunits of CT has been seen to affect its biological activity and the interaction with proteins. A previous work studied synthesized CT dimers and trimers and found that the number of active sites that were able to bind proteins increased with the number of cis flavanol units [33]. However, to date, literature contains very little information is available on the role of CT cis/trans ratio in terms of the biological activity. Therefore, the rapid and accurate determination of both ratios by MIR can help not only to optimally select tree individuals among the species at the right moment but also increasing the knowledge regarding seasonal variability and animal biological activity of the CT features.

### Full condensed tannin profile model

Table 3 shows the regression performance for the joint tannin profiling of all the parameters (including HBAI as the method for quantification) analyzed by a single PLS-R model (see Additional file 3: Fig. S3). The HBAI was used as the reference method for quantifying tannins in the full model, taking into account the results obtained in the individual models and the described limitations of thiolysis for this purpose. The fingerprint region offered the best results, with the most relevant VIP values (> 2) (Additional file 2: Fig. S2) falling near wavelengths with absorption features associated with phenolic compounds (1080–1200 and 1465–1475 cm^−1^) and aromatic out-of-plane bending (670–900 cm^−1^), as detected before in the individual models. Overall, the prediction metrics (R^2^P and RMSEP) obtained by the joint model were slightly worse than the individual ones. However, except for mDP, the value of both parameters employed to assess the models’ practical utility value (RPD > 3 and RER > 10) remained above the threshold considered for excellent models. Besides, full model showed improved RMSECV/RMSEC ratio (≤ 1.20) in all parameters, indicating higher robustness.

**Table 3:** PLS-R parameters for the tannin profile analyzed by a single model

	%CT (HBAI)	mDP	PC	cis
LV	8			
N	Calibration: 89/validation: 30			
Wavelengths (cm^−1^)	550–1800			
RMSEC	0.65	0.80	6.71	8.03
R^2^C	0.91	0.59	0.94	0.94
RMSECV	0.76	0.96	8.14	9.62
RMSEP	0.81	0.85	7.23	8.82
R^2^P	0.90	0.68	0.94	0.92
RPD	3.21	1.69	4.07	3.39
RER	11.3	6.49	10.9	10.6

*%CT* content of condensed tannins (%DM) measured by HCl-butanol-acetone-iron assay (HBAI), *mDP* mean degree of polymerisation, *PC* % procyanidin units (% condensed tannins), *cis* % cis units (% condensed tannins), *LV* latent variables, *N* number of samples, *RMSEC/CV/P* root-mean-square error or calibration/cross-validation/prediction, *RPD* ratio of prediction to deviation, *RER* range error ratio

Consequently, the full model’s application allows a rapid characterization of the most relevant CT parameters (CT%, PC/PD and cis/trans) of willow, oak and maple foliage samples with sufficient precision and robustness. This fact could represent an advantage compared to individual models for screening purposes of large amounts of samples. In contrast, if more in-depth information about a specific characteristic of the CT profile is desired, the individual models might be a better choice, according to the improved metrics.

The development of this quick method for a comprehensive analysis of CT profile opens the door to a two-way evaluation of tree foliage for ruminant nutrition purposes. First, the employment of current knowledge on tannins to assess the optimum stages of individual trees to be used for animal nutrition would assist in decision-making in silvopastoral systems. Secondly, and possibly more relevant, to understand better the effect of environmental conditions on tannin profile variations and the effect of this variability on animal health and performance. Due to time and money limitations of the reference laboratory methods (thiolysis/HBAI), CT profiling of tree foliage is not routinely performed, and much information regarding CT variability remains unknown. The periodical monitoring of the full profile of large quantities of samples would permit the obtention of a high amount of data, allowing a better understanding of CT physiology and the consequences on animal nutrition, despite the inevitable sacrifice in accuracy compared to the reference methods.

## Conclusions

The present study demonstrates the possibility of quickly obtaining a full CT profile of the dried tree foliage with a high level of robustness and precision in relation to the reference methods employed. The simultaneous and repeated screening of full CT profile from different plant species in a large number of samples represents a strong argument for the use of reflectance spectroscopy in silvopastoral systems. The information obtained would help to understand better plant ecology aspects (genetic variation, response to weather conditions, grazing) and ultimately improve the nutritional management of ruminant livestock. In contrast with previous attempts using NIR calibration models, the present study clearly demonstrated the efficacy of MIR combined with multivariate data analysis to accurately determine the tannin concentration and structure of oak, field maple and willow foliage. However, to make full use of what these methods can offer in silvopastoral systems, in situ*-*in vivo field development using portable equipment would be required. To fulfil this objective, a broader range of samples should be included to obtain robust models that allow compensating the potential loss of accuracy related to measurements performed in fresh foliage.

## Methods

### Sample collection

Samples of leaves and twigs were collected in a dry state from three tree species; oak (*Quercus robur*), field maple (*Acer camprestre*), goat willow (*Salix caprea*) from Elm Farm, an 85 ha organic livestock farm in West Berkshire (51° 23′ 14.19″ N; 1° 24′ 08.34″ W), with soil types varying from heavy clay loam to sandy loam (Eutric Luvic Planosols). For each species, five individual trees were sampled monthly from June to September 2017. Sampled trees were spread across the farm and either in boundary hedges or in-field. To obtain the leaf and twig samples, branches with a maximum diameter of 10 mm were selected from four orientations (North, East, South, West) around the tree crown and cut using secateurs. A sample of 600 g of fresh leaves and 600 g of fresh twigs were collected by separating the leaves and petioles from the lopped branches by gloved hand into paper bags. Samples were then oven-dried at 40 °C until a constant mass was achieved and milled at ≤ 1 mm particle size.

### Condensed tannins analysis

Total condensed tannins were quantified according to Grabber and Zeller [7] and their profile was assessed using the methods of Gea et al. [34]. Quantitative data refer to the content of extractable plus unextractable CT; whereas qualitative data provide information on tannin structures, such as, mean degree of polymerization (mDP) (i.e., molecular size), prodelphinidin:procyanidin (PD:PC) (i.e., PD value) and cis:trans (i.e., cis value) ratios.

### Collection of the MIR spectra

Leaf and twigs samples were freeze-dried, ground and passed through a 0.02 mm (mesh 70) sieve (Glenammer Sieves Ltd., UK). MIR spectra were acquired at ambient temperature (20 ± 2 °C) using a Nicolet iS50 FT-IR spectrometer Thermo Nicolet iS50 coupled with an ATR iD7 accessory (Thermo Fisher Scientific, Dublin, Ireland). The instrument uses a typical diamond crystal, ZnSe lens, and DTGS KBr detector. More specifically, after the sample holder was cleaned with alcohol and dust-free tissue, the samples were placed on the flat surface of the crystal while the slip clutch tower applied equal pressure. Thirty-two scans per sample were collected in the mid-infrared range from 550 to 4000 cm^−1^ in transmission mode at a spectral resolution of 4 cm^−1^. The spectra were corrected against air as background. Three different replicates of each sample were measured and averaged before data preprocessing. Each IR spectrum was normalized and the baseline corrected with OMNIC 7.3 software (Spectra-Tech Inc., Madison, WI, USA).

### Regression model development

Linear regression models using Partial Least Squares Regression (PLS-R) calculated with the SIMPLS algorithm were developed for tannin profiling quantification [35]. First, one individual model was built for each parameter (CT%, mDP, PC:PC ratio and cis:trans ratio); finally, a full model was built to simultaneously quantify all parameters of interest for CT profiling. Spectral preprocessing and multivariate data analysis were performed with PLStoolbox (version 8.8.1, Eigenvector Research Inc., Manson, WA, USA). Tree foliage samples were divided into a calibration set (75% of the samples) and an external validation set (25% of the samples). Assignment of samples to either the calibration or validation set was randomized by assigning each sample a number using the RANDOM function in MS Excel, taking into account the different groups included in the sample set. Three different calibration:validation sets were randomly constructed; results are showing the average value of the different parameters. Table 1 shows a descriptive summary of the calibration and validation sets statistics, both with an appropriate level of variation representative of the whole data set. Prior to calibration, data matrices were preprocessed applying in sequential order: mean-centering, SNV and a weighed Savitsky-Golay filter (polynomial degree = 2; window size = 11 points), which implied the application of 2nd derivative and smoothing [36].

Full spectra (550–4000 cm^−1^), partial (principal absorption bands), and optimized local PLS models were assessed. Local PLS models were optimized using interval partial least-squares (iPLS) and auto-VIP/sRatio in PLSToolbox, methods which search for the most informative regions in the spectra with important covarying spectral regions to build models on fewer variables. PLS models with 1–10 latent variables (LVs) were investigated. The optimal number of PLS variables to use in the PLS models was obtained by the cross-validation method with the Venetian blinds option (7 data splits with 1 sample per blind). The determination was performed visually by assessing the cut-off point in which no significant improvement (< 5%) of root mean square error of cross-validation (RMSECV) was obtained without increasing RMSECV/root mean square error of calibration (RMSEC) ratio to avoid overfitting. Moreover, the practical predictive ability of the PLS models was further assessed by the external prediction set. During the calibration and prediction procedure, potential outliers were detected by the Leverage and Q residuals values in sample sets. A shortlist of potential outliers was elaborate when leverage or Q residuals values exceeded the corresponding limits (Leverage ≥ (3 × nºLVs)/nº samples; α_Q_ > 0.05) [37] and the model performance was improved after their substraction. Following, shortlisted outliers were re-analysed by FT-MIR to check the quality of the spectra acquisition and registers of the sampling at the field were inspected. Finally, < 2% of outliers were identified and removed from the models, as indicated in Tables 1 and 3.

The developed models were evaluated in terms of: (a) coefficient of determination for Calibration (R^2^C), cross-validation (R^2^CV) and prediction (R^2^P); (b) root mean square error of calibration (RMSEC), cross-validation (RMSECV) and prediction (RMSEP) and (c) prediction bias. Also, the ratio of prediction to deviation (RPD = SD/RMSEP), and the range error ratio (RER = Range/RMSEP) were calculated to assess the practical utility value of the models [38]. The best regression equations were selected by optimizing the following combinations: minimize RMSECV and RMSEP, and maximize coefficients of determination, RPD and RER values. The classification of the model performance was performed based on Williams [38] and Fearn [39] as excellent (R^2^ ≥ 0.95, RPD ≥ 3, RER ≥ 10); modest (R^2^ = 0.80–0.94, RPD = 2–3, RER = 3–10); or weak (R^2^ < 0.80, RPD < 2, RER < 3). Finally, the contribution of PLS-R loadings by individual wavelengths was further determined using the Variable In Projection (VIP) method [40] (Additional file 4).

## Supplementary Information

**Additional file 1: Figure S1.** Principal Component Analysis of leaves and twigs samples of willow, oak and field maple.

**Additional file 2: Figure S2.** Variable importance of FT-MIR spectra to phytochemical calibrations for oak, field maple and willow (VIP analysis).

**Additional file 3: Figure S3.** Actual vs. predicted values of the full-model tannin profile parameters. Grey spots: calibration samples; Red diamond: validation samples; Green line: ideal prediction fit; Red line: predicted adjusted equation; R^2^P: coefficient of prediction.

**Additional file 4: Table S1.** Summary of Calibration and Prediction PCR parameters for the tannin profile. **Table S2.** Summary of Calibration and Prediction MLR parameters for the tannin profile.

## Data Availability

The datasets generated during and/or analysed during the current study are not publicly available as part of the data belong to a wider ongoing project with pending publications on the topic, but are available from the corresponding author on reasonable request.
