# Supplementary figures and images for: Rapid tannin profiling of tree fodders using untargeted mid-infrared spectroscopy and partial least squares regression
Source: Plant Methods. 2021 Feb 6;17:14. doi: 10.1186/s13007-021-00715-8 (PMC7866629; doi:10.1186/s13007-021-00715-8)

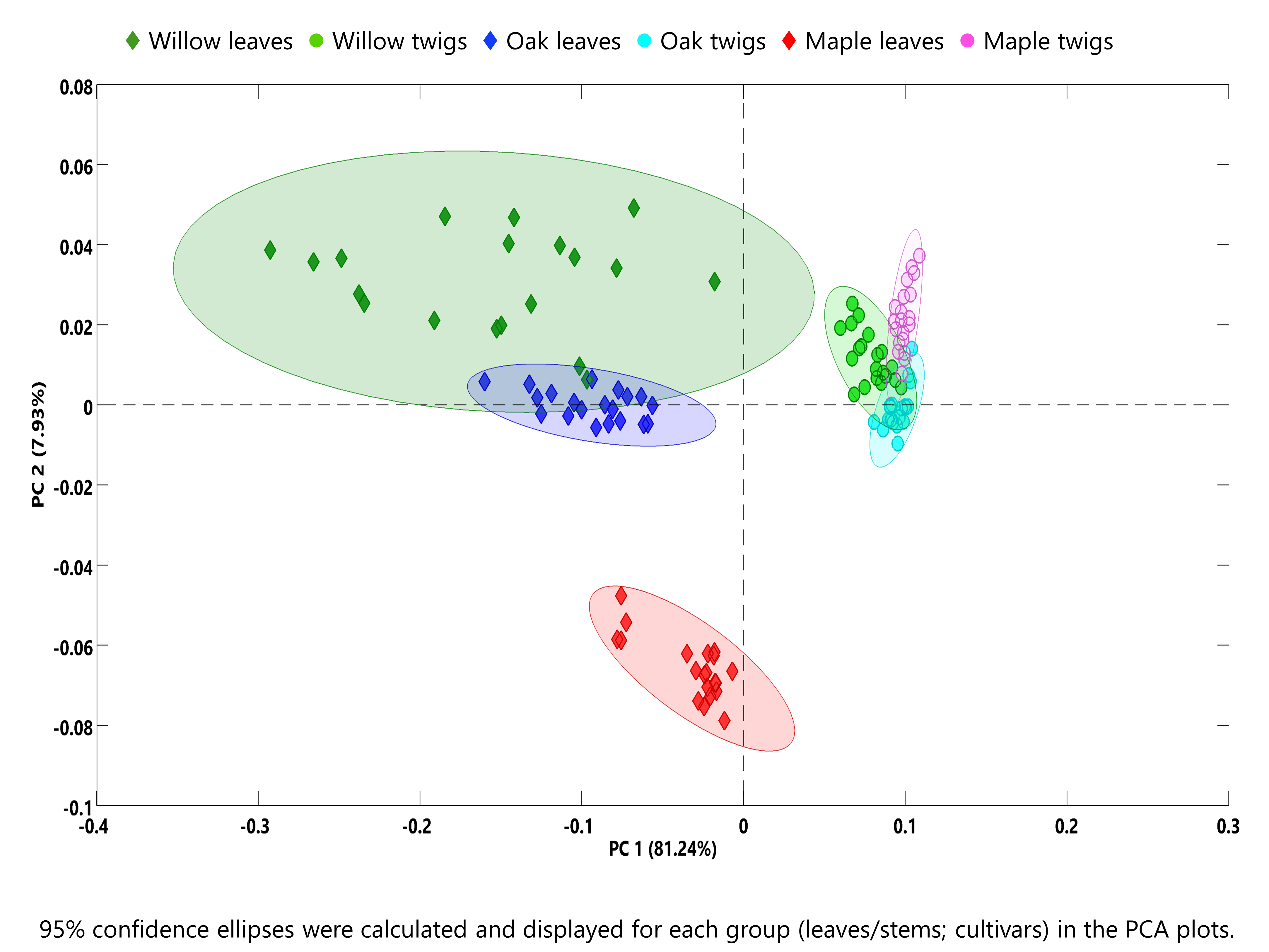

Supplement: Supplementary file 1 — Additional file 1: Figure S1. Principal Component Analysis of leaves and twigs samples of willow, oak and field maple. [file 13007_2021_715_MOESM1_ESM.tif]

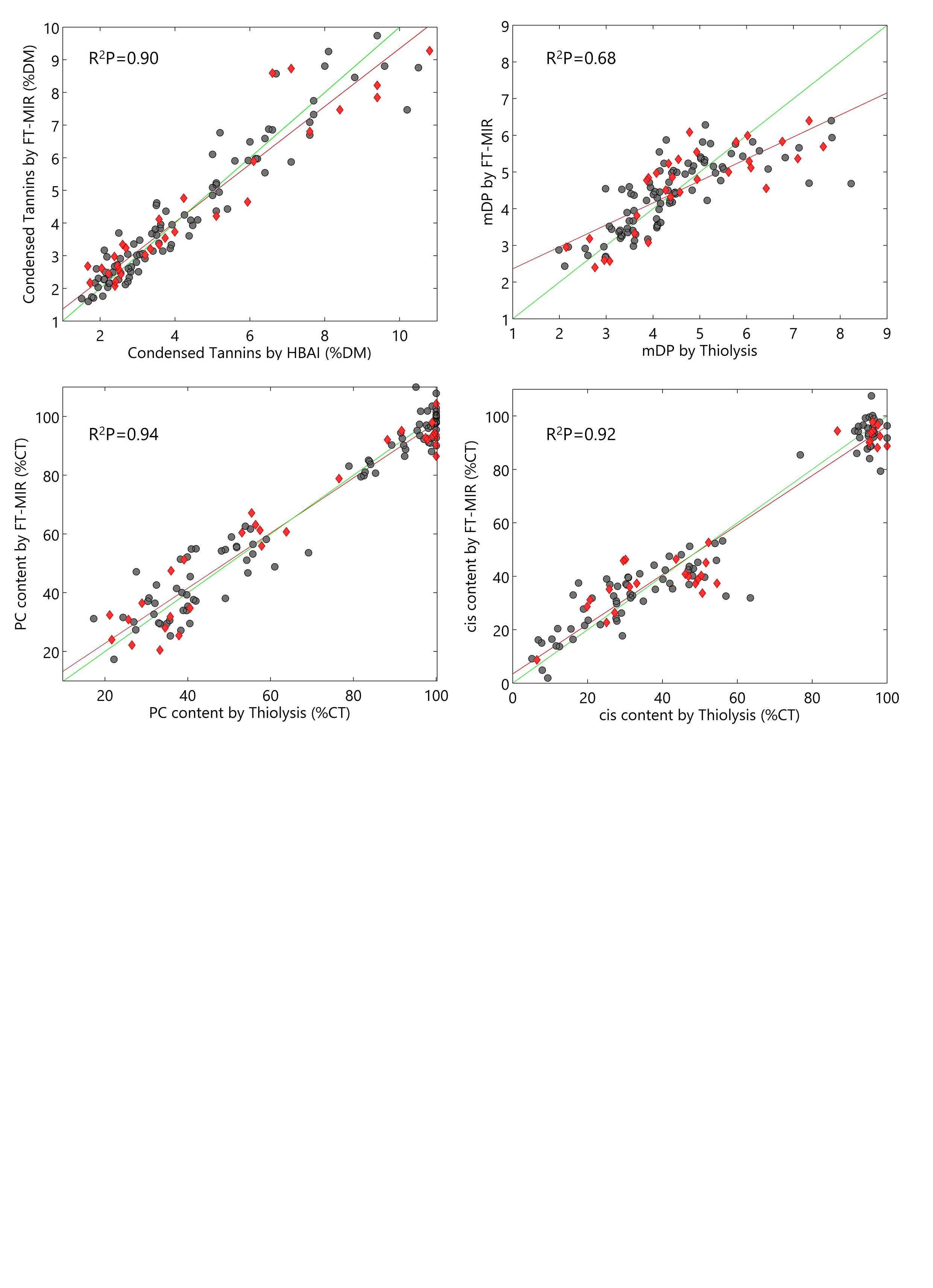

Supplement: Supplementary file 3 — Additional file 3: Figure S3. Actual vs. predicted values of the full-model tannin profile parameters. Grey spots: calibration samples; Red diamond: validation samples; Green line: ideal prediction fit; Red line: predicted adjusted equation; R2P: coefficient of prediction. [file 13007_2021_715_MOESM3_ESM.tif]
