# Supplementary material for: Rapid tannin profiling of tree fodders using untargeted mid-infrared spectroscopy and partial least squares regression
Source: Plant Methods. 2021 Feb 6;17:14. doi: 10.1186/s13007-021-00715-8 (PMC7866629; doi:10.1186/s13007-021-00715-8)

Condensed Tannins by HBAI

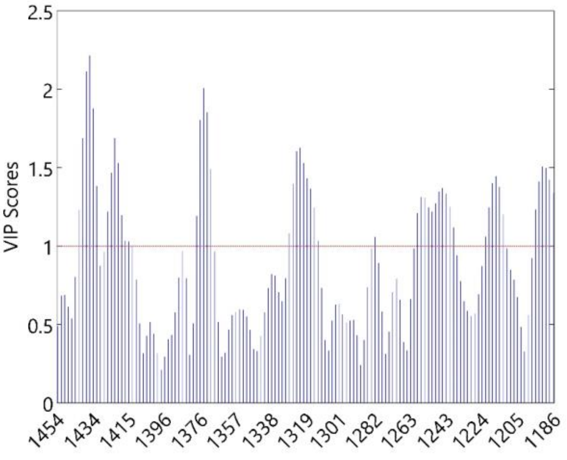

Mean Degree of Polymerization (mDP)

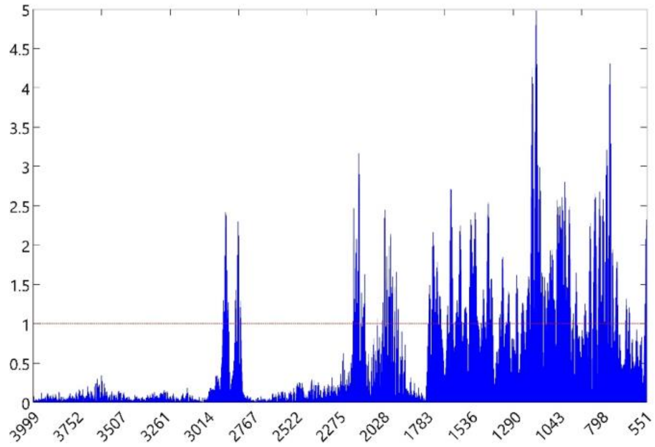

Condensed Tannins by thiolysis-HPLC

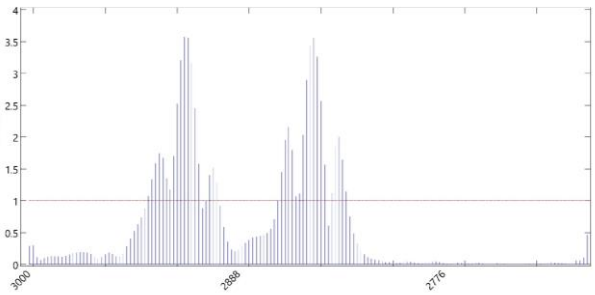

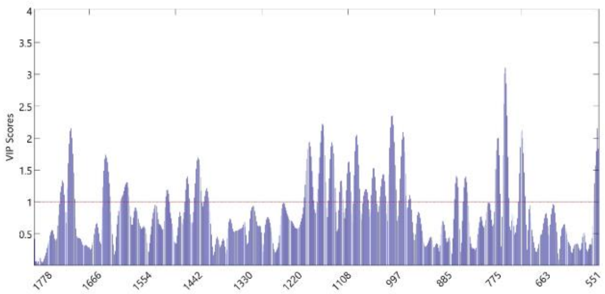

Procyanidin content (PC)

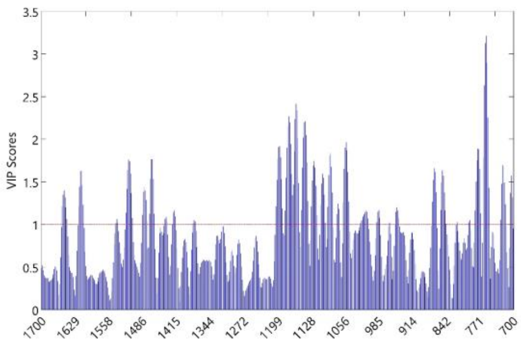

cis isomers content

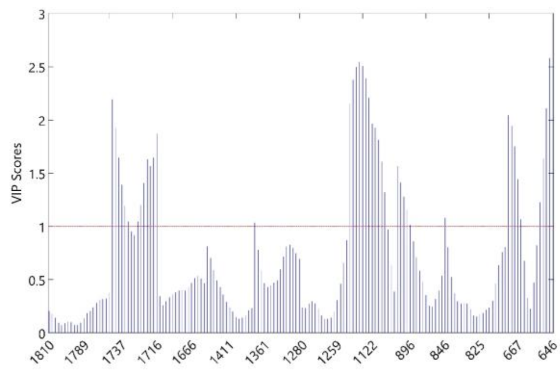

Supplement: Supplementary file 2 — Additional file 2: Figure S2. Variable importance of FT-MIR spectra to phytochemical calibrations for oak, field maple and willow (VIP analysis). [file 13007_2021_715_MOESM2_ESM.pdf]
