# Supplementary material for: Rapid tannin profiling of tree fodders using untargeted mid-infrared spectroscopy and partial least squares regression
Source: Plant Methods. 2021 Feb 6;17:14. doi: 10.1186/s13007-021-00715-8 (PMC7866629; doi:10.1186/s13007-021-00715-8)
Supplement: Supplementary file 4 — Additional file 4: Table S1. Summary of Calibration and Prediction PCR parameters for the tannin profile. Table S2. Summary of Calibration and Prediction MLR parameters for the tannin profile. [file 13007_2021_715_MOESM4_ESM.pdf]

Table S1. Summary of Calibration and Prediction PCR parameters for the tannin profile

	PCs	Regions (cm^{-1})	R^2C	RMSEC	RMSECV	R^2P	RMSEP
**%CT** (HBAI)	7	iPLS (1186-1454)	0.82	0.92	1.04	0.86	1.01
**%CT** (Thiolysis)	4	Main frequencies (551-1810/ 2700-3000)	0.56	1.62	1.76	0.55	1.88
**mDP**	3	Full range (550-4000)	0.31	1.03	1.12	0.48	1.13
**PC**	8	Fingerprint (700-1700)	0.87	10.2	12.8	0.88	10.8
**cis**	6	iPLS (9 regions[b])	0.94	8.27	9.16	0.90	9.62

Table S2. Summary of Calibration and Prediction MLR parameters for the tannin profile

	Regions (cm^{-1})	R^2C	RMSEC	RMSECV	R^2P	RMSEP
**%CT** (HBAI)	iPLS (1186-1454)	1	$1.61x10^{-13}$	1.43	0.76	1.32
**%CT** (Thiolysis)	Main frequencies (551-1810/ 2700-3000)	0.999	0.17	1.58	0.75	1.56
**mDP**	Full range (550-4000)	1	$1.35x10^{-14}$	1.09	0.41	1.63
**PC**	Fingerprint (700-1700)	1	$1.18x10^{-12}$	8.80	0.96	6.26
**cis**	iPLS (9 regions[b])	1	$1.42x10^{-10}$	17.8	0.74	18.9
